# High glucose contributes to the proliferation and migration of non-small-cell lung cancer cells via GAS5-TRIB3 axis

**DOI:** 10.1042/BSR20171014

**Published:** 2018-03-16

**Authors:** Cheng-Zhi Ding, Xu-Feng Guo, Guo-Lei Wang, Hong-Tao Wang, Guang-Hui Xu, Yuan-Yuan Liu, Zhen-Jiang Wu, Yu-Hang Chen, Jiao Wang, Wen-Guang Wang

**Affiliations:** 1 Department of Thoracic Oncology, Henan Provincial Chest Hospital, Zhengzhou, China; 2 Department of Thoracic Surgery, Shanghai Chest Hospital, School of Medicine, Shanghai Jiaotong University, Shanghai, China; 3 Department of Thoracic Surgery, Henan Provincial Chest Hospital, Zhengzhou, China; 4 Division of Endocrinology, Department of Internal Medicine, The First Affiliated Hospital of Zhengzhou University, Zhengzhou, China

**Keywords:** GAS5, high glucose, migration, NSCLC cells, proliferation, TRIB3

## Abstract

Despite the growing number of studies exhibiting an association of diabetes mellitus (DM) and lung cancer progression, the concrete mechanism of DM aggravating lung cancer has not been elucidated. The present study was to investigate whether and how high glucose (HG) contributes to the proliferation and migration of non-small-cell lung cancer (NSCLC) cells *in vitro*. In the present study, we confirmed that HG promoted the proliferation and migration of NSCLC cells, and also induced an anti-apoptotic effect on NSCLC cells. Moreover, HG inhibited the expression of growth arrest-specific 5 (GAS5) in NSCLC cells but elevated the protein level of tribbles homolog 3 (TRIB3). GAS5 overexpression promoted the degradation of TRIB3 protein by ubiquitination and inhibited the HG-induced proliferation, anti-apoptosis, and migration of NSCLC cells. Importantly, TRIB3 overexpression reversed the effects of GAS5 on the HG-treated NSCLC cells. Taken together, down-regulated GAS5 by HG significantly enhanced the proliferation, anti-apoptosis, and migration in NSCLC cells through TRIB3, thus promoting the carcinogenesis of NSCLC.

## Introduction

Lung cancer is one of the most common malignant tumors with the highest morbidity and mortality that seriously threatens human health and life [1]. Non-small-cell lung cancer (NSCLC) is the most common histological type of lung cancer, which accounts for ~80% of all lung cancers [2]. Because most patients have been diagnosed at advanced stage, the 5-year survival rate in NSCLC patients is very low. NSCLC may result from comprehensive action of multiple factors and the pathogenesis remains largely unknown.

Insulin resistance occurred in early diabetic patients and induced metabolic derangements, including glycometabolism and lipid metabolism, which are associated with the pathologic processes of cardiovascular diseases, hypertension, and cancers [3–5]. As the high-risk factor for malignant tumors, the mortality rate of tumor patients with diabetes mellitus (DM) was significantly higher than those without DM. It was found that DM was directly related to the poor prognosis of patients with lung cancer, especially the NSCLC patients treated by surgery [6,7]. Hyperglycemia, insulin resistance, hyperinsulinemia are the typical metabolic disorders of DM, but it is uncertain whether they are the critical events that induce cancer development. In recent years, some mechanisms have been hypothesized to explore how an excess level of glucose affected the occurrence and development of tumors, such as breast, colorectal, pancreatic, and lung cancer [8–10]. High gluose (HG) can promote the proliferation, migration, and invasion of cancer cells through various mechanisms. Despite the growing number of studies exhibited an association of HG and lung cancer progression, the concrete mechanism of HG promoting lung cancer has not been elucidated.

Long non-coding RNA (lncRNA) is a class of non-protein coding RNA transcripts with a length greater than 200 nts. Studies have shown that lncRNA was involved in transcription, post-transcriptional, and epigenetic mechanisms that regulated a variety of pathophysiological processes of the body. Multiple lncRNAs were confirmed to be involved in the context of lung cancer [11], including lncRNA growth arrest-specific 5 (GAS5). It has been reported that GAS5 expression was decreased in cancerous tissues of NSCLC patients as a tumor suppressor; GAS5 suppressed tumorigenesis in NSCLC *in vitro* and *in vivo* [12,13]. More significantly, GAS5 levels were distinctly decreased in serum of DM patients and correlated to prevalence of DM [14]. Therefore, it was interesting that whether GAS5 could be the bridge factor between HG and lung cancer.

Pseudokinase tribbles homolog 3 (TRIB3) was first found in *Drosophila* and could inhibit the mitosis in germ cells [15]. Further studies confirmed that it was expressed in a variety of human cells that played ubiquitous cellular functions in proliferation, apoptosis, and adhesion [16]. A recent study identified that TRIB3 was up-regulated in NSCLC that promoted the metastasis and tumor growth [17]. Furthermore, TRIB3 has a pivotal role in insulin signaling transduction pathway [18], and the DM-induced tumor progression [19]. Thus, it is reasonable to infer that TRIB3 may participate in the development of NSCLC induced by HG. Given the above, the aim of the present study was to investigate the role of GAS5 and TRIB3 in HG-induced NSCLC and to reveal the possible interactions between GAS5 and TRIB3.

## Materials and methods

### Cell culture and teatment

The NSCLC cell lines (PC-9 and H1299) were purchased from the Chinese Academy of Sciences (Shanghai, China) and cultured in Dulbecco’s modified Eagle’s medium (DMEM) (HyClone) containing HG (25 mM) or low glucose (LG; 5 mM) and 10% FBS (Gibco), 100 U/ml penicillin, and 100 μg/ml streptomycin at 37°C in 5% CO_2_. HG treatment for 24 h was used to induce the model of DM combined with NSCLC *in vitro* [20].

### Quantitative RT-PCR

Quantitative RT-PCR (qRT-PCR) was used for detection of the target molecules expression at mRNA level. The cells were treated with TRIzol (Invitrogen) to obtain total RNA according to the manufacturer’s instructions. The total RNA was then reverse transcribed into cDNA using the PrimeScript™ RT reagent kit (Takara) according to the manufacturer’s instructions. The cDNA product was further amplified on an ABI 7500 system (Applied Biosystems) with the SYBR Premix Ex Taq (TaKaRa). The specific primers for GAS5 and TRIB3 were provided by Sangon Biotech (Shanghai) Co., Ltd. (Shanghai, China). *GAPDH* mRNA was used for the internal control and the relative expressions of GAS5 and TRIB3 were calculated using the 2 ^–ΔΔ*C*^_t_ method. The specific primer sequences were as follows: GAS5, (forward) 5-CTCTGGATAGCACCTTATGGAC-3 and (reverse) 5-CTTCCAGCTTTCTGTCTAATGCC-3; TRIB3, (forward) 5-GCCTTTTTCACTCGGACCCAT-3 and (reverse) 5-CAGCGAAGACAAAGCGACAC-3; GAPDH, (forward) 5-CTCTGACTTCAACAGCGACACC-3 and (reverse) 5-CTGTTGCTGTAGCCAAATTCGTT-3.

### Western blot

The expression of TRIB3 protein was measured using Western blot analysis performed according to the previously described standard procedures [21]. Total protein (25 μg/sample) was separated by SDS/PAGE and then transferred on to PVDF (Millipore) membranes. The membranes were blocked with 5% skim milk in 0.1% Tween 20 for 1 h at room temperature. The proteins were incubated with the specific primary antibodies at 4°C overnight and then incubated with the horseradish peroxidase conjugated-secondary antibodies for 2 h at room temperature. Anti-TRIB3 antibodies (1:1000) and anti-β-actin antibodies (1:2000) were purchased from Abcam and Cell Signaling Technology, respectively. The horseradish peroxidase conjugated-secondary antibodies (1:1000) were purchased from Abcam. The protein bands were visualized by ECL reagents (ThermolBiotech) and the relative density of bands was also quantitated by ImageJ software (U.S.A.).

### Cell transfection

The plasmid vectors containing GAS5/TRIB3 sequence (pcDNA-GAS5/TRIB3) were constructed by (Jie Li Biology, China). The specific RNA interfere sequences (5′-GCGAGCGCAATGTAAGCAA-3′) for GAS5 (GAS5 siRNAs) were purchased from RiboBio (Guangzhou, China). NSCLC cells (2 × 10^5^ cells/well) were cultured in six-well plates for 24 h. The plasmid vectors or GAS5 siRNAs were transfected into NSCLC cells by using Lipofectamine 2000 Transfection Reagent (Invitrogen) according to the manufacturer’s protocols. The working concentration of GAS5 siRNAs was 20 nM.

### RNA pull-down assay

The biotin-labeled RNA sequences were obtained by using Biotin RNA Labeling Mix (Roche) and RNeasy Plus Mini Kit (Qiagen). According to the previous protocol [22], 1 mg protein from H1299 cells were mixed with the biotin-labeled RNA (3 μg) and then incubated with the magnetic beads (50 µl) (Invitrogen) for 2 h at 4°C. The retrieved protein was collected by Biotin Elution Buffer and further detected with Western blot analysis as mentioned above.

### RIP assay

RIP assay was carried out with the RNA Binding Protein Immunoprecipitation Kit (Merck Millipore) according to the manufacturer’s instructions. Briefly, anti-TRIB3 antibody and anti- IgG antibody (Cell Signaling Technology) were incubated with the sepharose beads (Milipore), respectively. The lysates of NSCLC cells were centrifuged to obtain the supernatants and the supernatants then incubated with the sepharose beads for 5 h at 4°C. Then, the RNA–protein complexes were isolated from beads by using SDS/Proteinase K. qRT-PCR was used for detection of the level of GAS5 in the protein–RNA complex precipatated by TRIB3.

### Cell viability assay

After 24 h of transfection, the cell viability of NSCLC cells was assessed by Cell Counting Kit-8 (CCK-8) (Beyotime Biotechnology) according to the manufacturer’s protocol. NSCLC cells (6.0 × 10^3^ cells/well) were cultured in 96-well plates and CCK-8 solution (10 μl) was added to each well of the plates. After incubation for 4 h at 37°C, the absorbance of each well was measured at 450 nm on a microplate reader. The relative viability of the cells was calculated using the following formula: relative viability = [(experimental OD − blank OD)/( control OD − blank OD)] × 100%.

### Cell migration assay

Transwell with 8.0 μm pores (Corning) was used for the cell migration assay in the present study. NSCLC cells (4 × 10^4^ cells) with different treatments were cultured in the upper chamber of transwells and the lower chamber contained complete medium. After culturing for 24 h at 37°C, the migrated cells were stained with Crystal Violet (0.1%) for 20 min. The numbers of the migrated cells were counted by using inverted microscope (Olympus).

### Cell cycle and cell apoptosis assay

Flow cytometry was performed to assess the effect of HG, GAS5, and TRIB3 on the cell cycle and apoptosis of NSCLC cells. The NSCLC cell lines (PC-9 and H1299) were divided into five groups: LG, HG, HG + pcDNA, HG + pcDNA-GAS5, and HG + pcDNA-GAS5 + pcDNA-TRIB3. NSCLC cells were transfected with pcDNA-GAS5 and/or pcDNA-TRIB3 as described above. After culturing under the LG or HG condition for 24 h at 37°C, the cells were harvested and fixed with ethanol (70%). For cell cycle analysis, the cells were treated with RNase A (Roche) and propidium iodide (PI, Roche) for 0.5 h in the dark. The cell cycle was detected on a Cytomics FC 500 flow cytometer (Beckman Coulter). For cell apoptosis analysis, the harvested cells were stained by fluorescein annexin-V and PI for 10 min and then analyzed on a Cytomics FC 500 flow cytometer. FlowJo software (U.S.A.) was used to analyze the cell cycle and cell apoptosis. The experiments were repeated three times.

### Detection of TRIB3 ubiquitination status

The NSCLC cell lines (PC-9 and H1299) were co-transfected with pcDNA-GAS5, My-TRIB3, HA-ubiquitin (Ub) and then treated with MG132 (10 μM) for 6 h. For immunoprecipitation, the Protein A/G beads (Life Technologies) coated with the indicated primary antibody were incubated with the cell extracts for overnight at 4°C. The precipitated proteins were eluted in low-salt buffer and analyzed by Western blot as described above.

### Statistical analysis

All statistical analysis in the present study were conducted by using SPSS 18.0 software package (IL). The statistical significance between groups was estimated by Student’s *t* test. *P*<0.05 was considered statistically significant in the current study. The data that came from three independent experiments were presented as the means ± S.D.

## Results

### HG induced the proliferation and migration of NSCLC cells through GAS5

**Figure 1:**
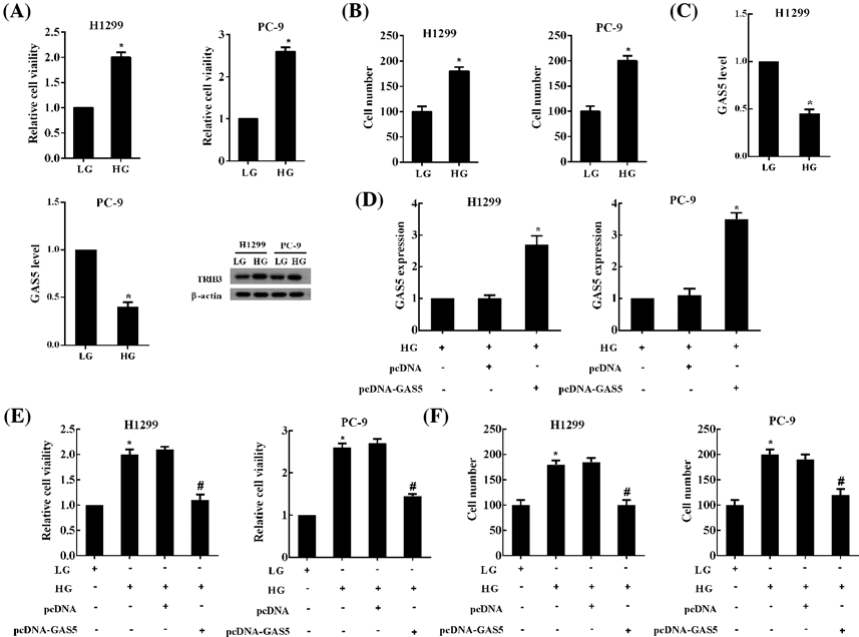
HG induced the proliferation and migration of NSCLC cells through GAS5 NSCLC cell lines (PC-9 and H1299) were cultured in LG (5 mM) and HG (25 mM) medium for 24 h, respectively. (**A**) The proliferation and (**B**) migration of NSCLC cells were detected using CCK-8 and Transwell assays, respectively. (**C**) The expression of GAS5 in NSCLC cell lines was determined by qRT-PCR, the levels of TRIB3 protein were measured using Western blot. NSCLC cells were transfected with pcDNA-GAS5 and then cultured with HG medium. (**D**) The expression of GAS5 in NSCLC cells was up-regulated by pcDNA-GAS5. GAS5 overexpression obviously suppressed the (**E**) proliferation and (**F**) migration of NSCLC cells treated with HG. For (A–D), *P<0.05 compared with LG or HG + pcDNA; for (E,F),*P<0.05 compared with LG,^#^*P*<0.05 compared with HG + pcDNA.

Compared with LG (5 mM) group, HG (25 mM) treatment for 24 h significantly increased the proliferation and migration of NSCLC cells (Figure 1A,B). However, the expression of GAS5 was significantly decreased in HG-treated NSCLC cells PC-9 and H1299 (Figure 1C). Moreover, the level of TRIB3 protein was dramatically increased in NSCLC cells cultured in HG medium (Figure 1C). HG induced the changes in the expression of two key molecules in NSCLC, suggesting that HG had a significant impact on the development of NSCLC. Further studies revealed that transfection of pcDNA-GAS5 in NSCLC cells disinhibited the GAS5 depression derived from HG treatment and GAS5 expression was significantly increased (Figure 1D). Moreover, GAS5 overexpression obviously suppressed the proliferation and migration of NSCLC cells treated with HG (Figure 1E,F).

### The interaction between GAS5 and TRIB3 protein in NSCLC cells

**Figure 2:**
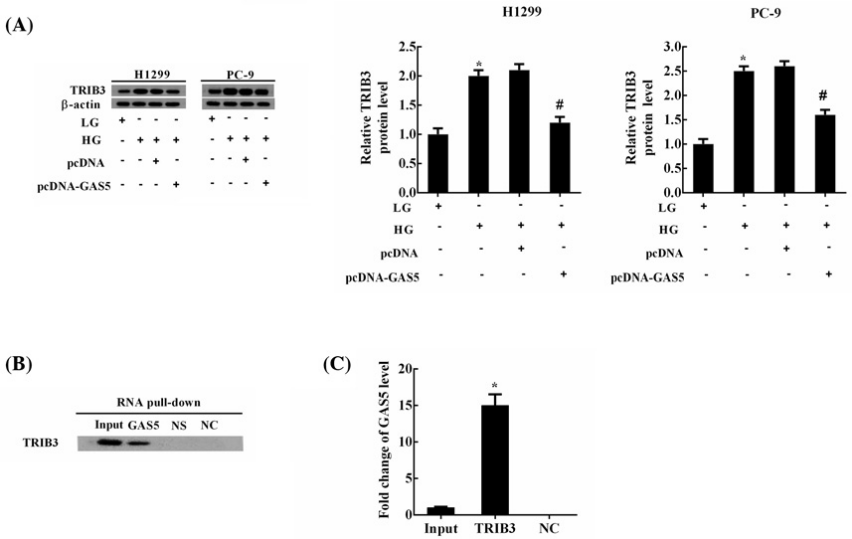
The interaction of GAS5 and TRIB3 in NSCLC cells NSCLC cells were divided into four experimental groups: LG, HG, HG + pcDNA (transfection of pcDNA and HG treatment) and HG + pcDNA-GAS5 (transfection of pcDNA-GAS5 and HG treatment). (**A**) The levels of TRIB3 protein were measured using Western blot; *P<0.05 compared with LG; ^#^*P*<0.05 compared with HG + pcDNA. (**B**) RNA pull-down assays revealed that TRIB3 protein was presented in the complex pull-down by GAS5. NS was the negative control for GAS5 and NC was the same RNA as GAS5, but it was without biotin label. (**C**) RIP assays confirmed that GAS5 was enriched in the protein–RNA complex precipatated by TRIB3 from NSCLC cells; ^#^*P*<0.05 compared with NC.

We have found that HG induced the proliferation and migration of NSCLC cells, at least in part, through regulating GAS5, so we further investigated that whether GAS5 mediated the effects of HG through regulating TRIB3 protein in NSCLC cells. HG significantly up-regulated the expression of TRIB3 protein, while this up-regulation decreased dramatically by GAS5 overexpression (Figure 2A). RNA pull-down assays revealed that TRIB3 protein was presented in the complex pull-down by GAS5 (Figure 2B). NS was the negative control for GAS5, and NC was the same RNA as GAS5, but it was without biotin label. RIP assays confirmed that GAS5 was enriched in the protein–RNA complex precipatated by TRIB3 from NSCLC cells (Figure 2C). All these results indicated that GAS5 could bind to TRIB3 protein.

### GAS5 promoted the degradation of TRIB3 protein in NSCLC cells

**Figure 3:**
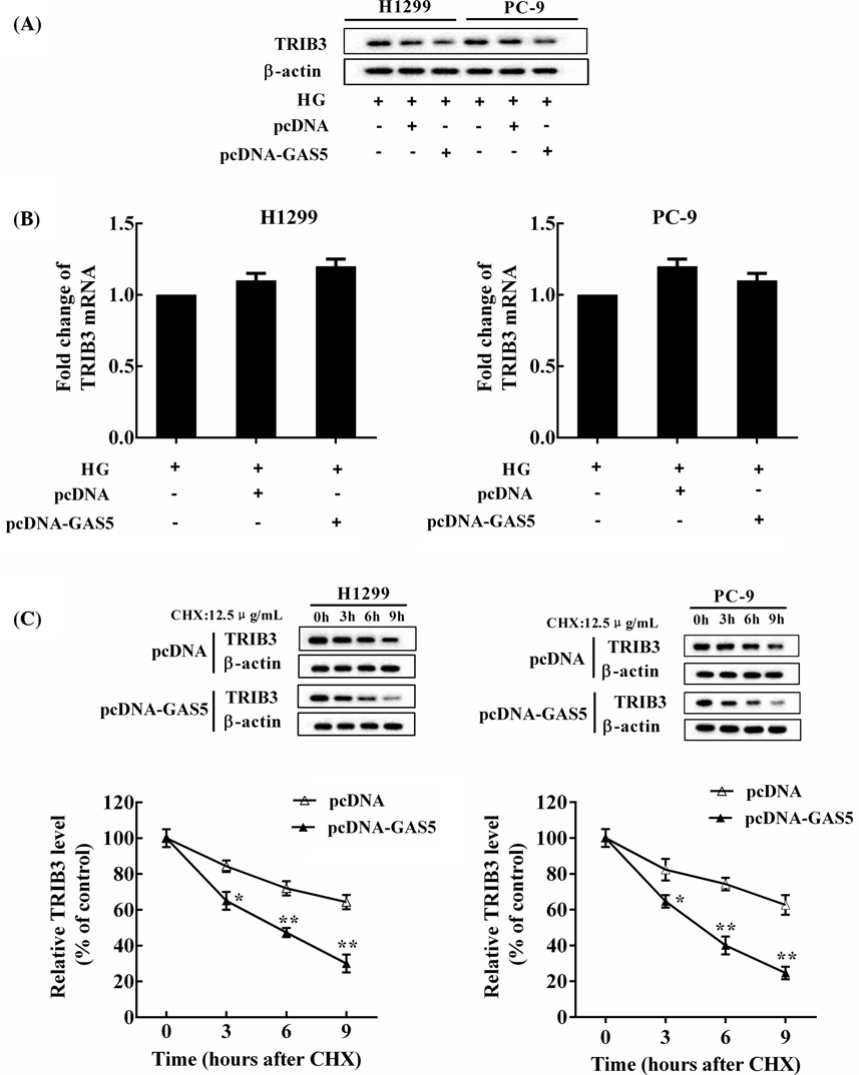
The level of TRIB3 protein was regulated by GAS5 NSCLC cells were cultured in HG (25 mM) and transfected with pcDNA-GAS5 and its negative controls, respectively. (**A**) TRIB3 protein was down-regulated by pcDNA-GAS5 in NSCLC cells. (**B**) GAS5 overexpression had no significant effect on the expression of *TRIB3* mRNA compared with the control. (**C**) NSCLC cells cultured in HG (25 mM) were transfected with pcDNA or pcDNA-GAS5 and then treated by CHX (12 μg/ml). The level of TRIB3 protein was measured at 0, 3, 6, 9 h after CHX treatment; *P<0.05, **P<0.01 compared with pcDNA.

TRIB3 protein was down-regulated by pcDNA-GAS5 in NSCLC cells (Figure 3A), while GAS5 overexpression had no significant effect on the level of *TRIB3* mRNA compared with the control (Figure 3B). NSCLC cells cultured in HG were transfected with pcDNA or pcDNA-GAS5 and then treated by cycloheximide (CHX, 12 μg/ml). GAS5 overexpression promoted the degradation of TRIB3 protein in NSCLC cells (Figure 3C). In addition, we employed the detection of TRIB3 ubiquitination status and Western blot analysis revealed that the endogenous TRIB3-associated ubiquitination was increased in NSCLC cells overexpressed GAS5, but the level of TRIB3 protein was decreased, indicating that GAS5 potentiated TRIB3 protein ubiquitination and subsequent degradation (Supplementary Figure 1).

### GAS5 inhibited the HG-induced proliferation, anti-apoptosis, and migration of NSCLC cells by regulating TRIB3 protein

**Figure 4:**
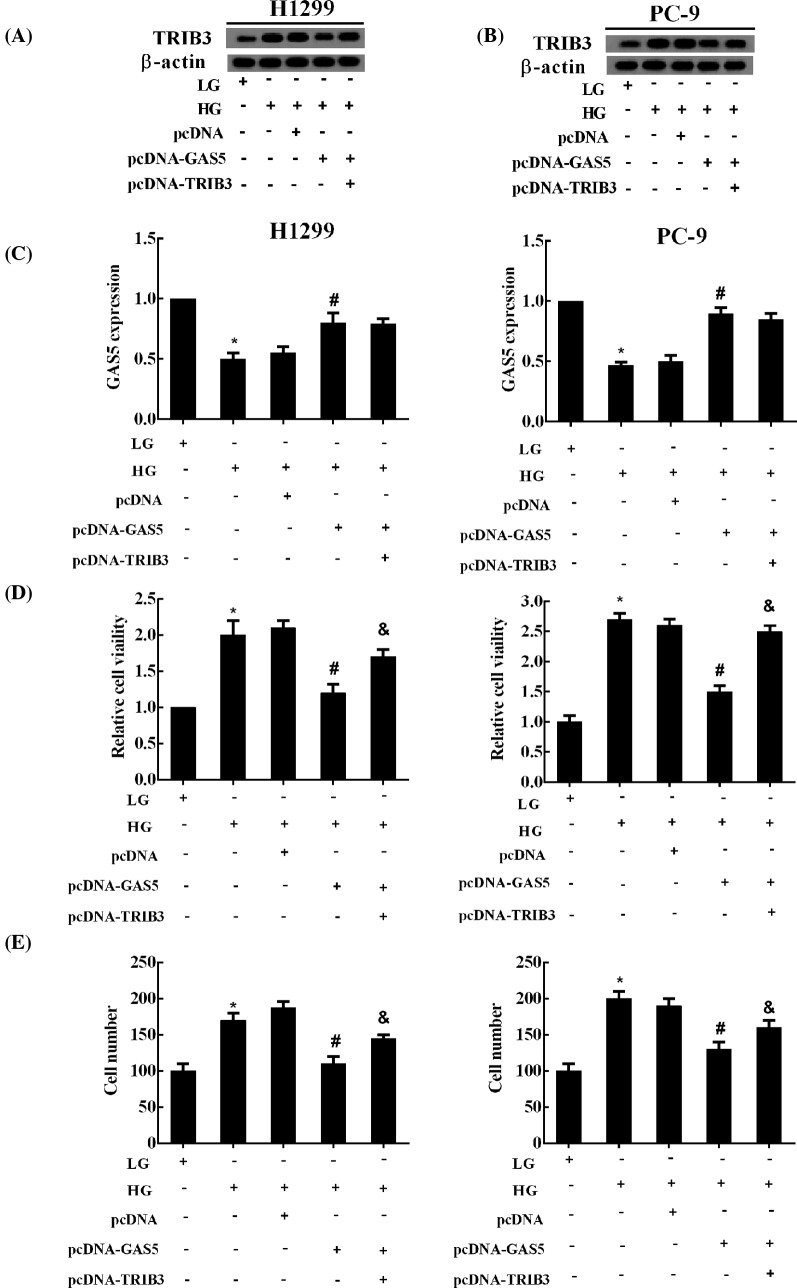
GAS5 inhibited the HG-induced proliferation and migration of NSCLC cells by regulating TRIB3 protein NSCLC cells were divided into five experimental groups: LG, HG, HG + pcDNA (transfection of pcDNA and HG treatment), HG + pcDNA-GAS5 (transfection of pcDNA-GAS5 and HG treatment), and HG + pcDNA-GAS5 + pcDNA-TRIB3 (co-transfection of pcDNA-GAS5 and pcDNA-TRIB3, and HG treatment). The expression of (**A**,**B**) TRIB3 protein and (**C**) GAS5 in NSCLC cells was measured using Western blot and qRT-PCR, respectively. The (**D**) proliferation and (**E**) migration of NSCLC cells was assessed by CCK-8 assay and Transwell system, respectively; *P<0.05 compared with LG; ^#^*P*<0.05 compared with + pcDNA; ^&^*P*<0.05 compared with HG + pcDNA-GAS5.

The pcDNA-GAS5-induced down-regulation of TRIB3 protein was reversed by pcDNA-TRIB3 (Figure 4A,B), while pcDNA-TRIB3 had no significant effect on the expression of GAS5 (Figure 4C). Furthermore, pcDNA-TRIB3 also reversed the pcDNA-GAS5-induced repression on the proliferation and migration of NSCLC cells (Figure 4D,E). Moreover, as shown in the Supplementary Figure 2A, cell cycle analysis showed that NSCLC cell number in G_1_-phase was decreased by HG treatment, but GAS5 was induced without altering cell cycle. Moreover, compared with LG, the apoptosis of NSCLC cells significantly decreased in HG group, and the forced GAS5 abolished the effect of HG, promoting NSCLC cell apoptosis (Supplementary Figure 2B). Furthermore, overexpressed TRIB3 combined with GAS5 up-regulation considerably reversed the GAS5-induced apoptosis of NSCLC cells, but it had no significance influence on cell cycle (Supplementary Figure 2A,B).

## Discussion

The HG in DM patients contributes to the initiation and progression of tumor [23]. In the present study, we confirmed that HG promoted the proliferation, anti-apoptosis, and migration of NSCLC cells *in vitro*. Moreover, HG inhibited the expression of lncRNA GAS5 in NSCLC cells but elevated the protein level of TRIB3. GAS5 could bind to TRIB3 protein to promote the degradation of TRIB3 protein and inhibited the HG-induced proliferation, anti-apoptosis, and migration of NSCLC cells. However, TRIB3 overexpression reversed the effects of GAS5 on the HG-treated NSCLC cells. These results identified that the carcinogenesis and progress of NSCLC induced by HG, at least in part, was mediated through GAS5-TRIB3 axis.

It has been reported that GAS5 expression was decreased in the cancerous tissues of NSCLC patients, as well as in the serum of DM patients [13,14]. GAS5 also has been found to induce cell apoptosis through enhancing cisplatin sensitivity in NSCLC [24]. In our study, GAS5 expression was decreased dramatically in NSCLC cells treated by HG. GAS5 overexpression could significantly inhibit the proliferation, invasion, and induced the apoptosis in NSCLC cells [12]. Therefore, we further investigated the role of GAS5 in the proliferation, apoptosis, and migration of NSCLC cells with HG and revealed that GAS5 was an important mediator of the HG-induced proliferation, anti-apoptosis, and migration in NSCLC cells. Cell cycle analysis showed that NSCLC cell number in G_1_-phase was decreased by HG treatment, but GAS5 was induced without altering cell cycle. Recent findings had revealed that the proliferation of NSCLC cells was independent of cell cycle position [25]. Furthermore, we also observed that HG elevated the protein level of TRIB3 in NSCLC cells. TRIB3 is a protein closely related to metabolism, and can be induced by a series of metabolism factors prevalent in DM, such as hyperglycemia, hyperinsulinemia, and IGF-1 [26,27]. TRIB3 was elevated in tumor lesions of patients with NSCLC which correlated with poor prognosis of NSCLC [17]. Furthermore, TRIB3 promoted the tumor development of liver and lung in DM mice [19]. Thus, we inferred that GAS5 might mediate the HG-induced progression of NSCLC by regulating TRIB3.

LncRNA was involved in the regulation of epigenetic modification, nuclear transport, transcription, and translation that played an important role in tumorigenesis [28]. In our study, GAS5 regulated the expression of TRIB3 at the protein level, but it did not influence the mRNA expression of TRIB3. By using CHX treatment, we confirmed that GAS5 negatively regulated the level of TRIB3 protein by promoting TRIB3 protein ubiquitination and subsequent degradation in NSCLC cells. LncRNAs were reported to recruit intracellular functional compounds and enzymes that regulated physiological and pathological progression [29]. We therefore speculated that GAS5 potentiated TRIB3 ubiquitination via modulating the interaction between ubiquitination or deubiquitinating enzymes and TRIB3. However, the concrete mechanisms underlying the regulation of GAS5 on TRIB3 ubiquitination need further investigation. Our current study also revealed that TRIB3 protein was regulated by GAS5 that mediated the GAS5-induced inhibition on the proliferation, anti-apoptosis, and migration of NSCLC cells treated by HG.

In summary, the present study provided strong evidence that lncRNA GAS5 acted as an important mediator of the HG-induced progression of NSCLC. Down-regulation of GAS5 by HG significantly enhanced the cell proliferation and migration in NSCLC cells, thus up-regulation of GAS5 was suggested to mitigate NSCLC in DM. From the present study, lncRNA GAS5 promises to be a target for the prevention and treatment of diabetic NSCLC.

## Supporting information

**supplemental Figure 1:** GAS5 potentiated TRIB3 protein ubiquitination. The NSCLC cell lines (PC-9 and H1299) were co-transfected with pcDNA-GAS5, My-TRIB3, HA-ubiquitin (Ub) and then treated with MG132 (10μM) for 6 hours. Western blot was performed to analyze the level of the endogenous TRIB3-associated ubiquitination and TRIB3 protein.

**supplemental Figure 2:** GAS5 attenuated the HG-induced anti-apoptosis of NSCLC cells without altering cell cycle. NSCLC cells were divided into 5 experimental groups: LG (low glucose treatment), HG (high glucose treatment), HG+pcDNA (transfection of pcDNA and high glucose treatment), HG+pcDNA-GAS5 (transfection of pcDNA-GAS5 and high glucose treatment) and HG+pcDNA-GAS5+pcDNA-TRIB3 (co-transfection of pcDNA-GAS5 and pcDNA-TRIB3, and high glucose treatment). (**A**) The cell cycle and (**B**) apoptosis of NSCLC cells were detected using Flow cytometry. *P<0.05 vs. LG; ^#^P<0.05 vs. HG + pcDNA; ^&^P<0.05 vs. HG + pcDNA-GAS5.

## Abbreviations

CCK-8: cell counting kit-8
CHX: cycloheximide
DM: diabetes mellitus
GAS5: growth arrest-specific 5
HG: high glucose
LG: low glucose
lncRNA: long non-coding RNA
NSCLC: non-small-cell lung cancer
PI: propidium iodide
qRT-PCR: quantitative Rt-PCr
TRIB3: tribbles homolog 3
