# Supplementary material for: High glucose contributes to the proliferation and migration of non-small-cell lung cancer cells via GAS5-TRIB3 axis
Source: Biosci Rep. 2018 Mar 16;38(2):BSR20171014. doi: 10.1042/BSR20171014 (PMC5857909; doi:10.1042/BSR20171014)
Supplement: Supplementary file 1 [file bsr20171014_Supp1.pdf]

c

supplemental Figure 1 GAS5 potentiated TRIB3 protein ubiquitination. The NSCLC cell lines (PC-9 and H1299) were co-transfected with pcDNA-GAS5, My-TRIB3, HA-ubiquitin (Ub) and then treated with MG132 (10μM) for 6 hours. Western blot was performed to analyze the level of the endogenous TRIB3-associated ubiquitination and TRIB3 protein.

supplemental Figure 2 GAS5 attenuated the HG-induced anti-apoptosis of NSCLC cells without altering cell cycle. NSCLC cells were divided into 5 experimental groups: LG (low glucose treatment), HG (high glucose treatment), HG+pcDNA (transfection of pcDNA and high glucose treatment), HG+pcDNA-GAS5 (transfection of pcDNA-GAS5 and high glucose treatment) and HG+pcDNA-GAS5+pcDNA-TRIB3 (co-transfection of pcDNA-GAS5 and pcDNA-TRIB3, and high glucose treatment). **(A)** The cell cycle and **(B)** apoptosis of NSCLC cells were detected using Flow cytometry. $^{*}P<0.05$ vs. LG; $^{\#}P<0.05$ vs. HG + pcDNA; $^{\&}P<0.05$ vs. HG + pcDNA-GAS5.

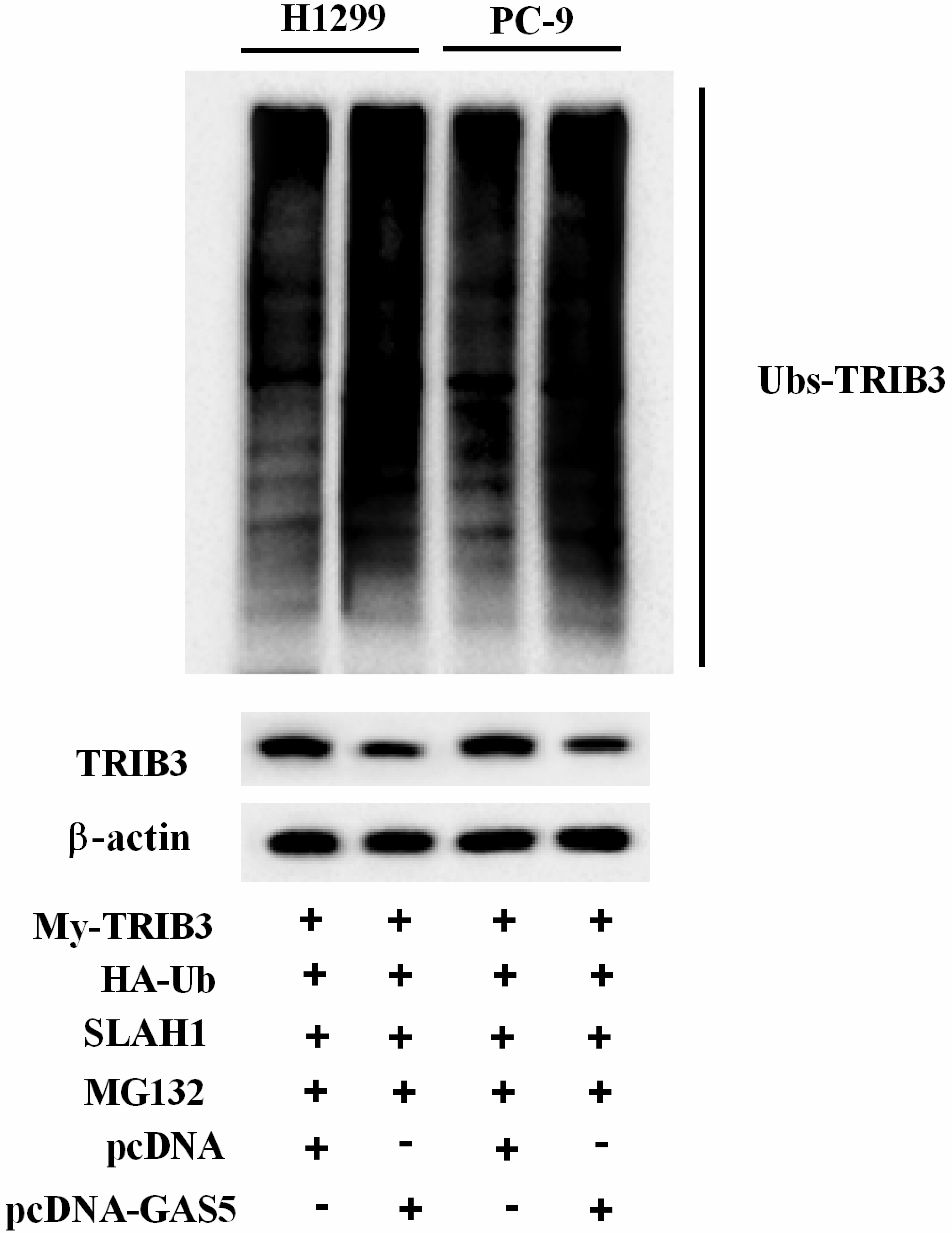
H1299
PC-9
Ubs-TRIB3
TRIB3
β-actin
My-TRIB3 + + + +
HA-Ub + + + +
SLAH1 + + + +
MG132 + + + +
pcDNA + - + -
pcDNA-GAS5 - + - +

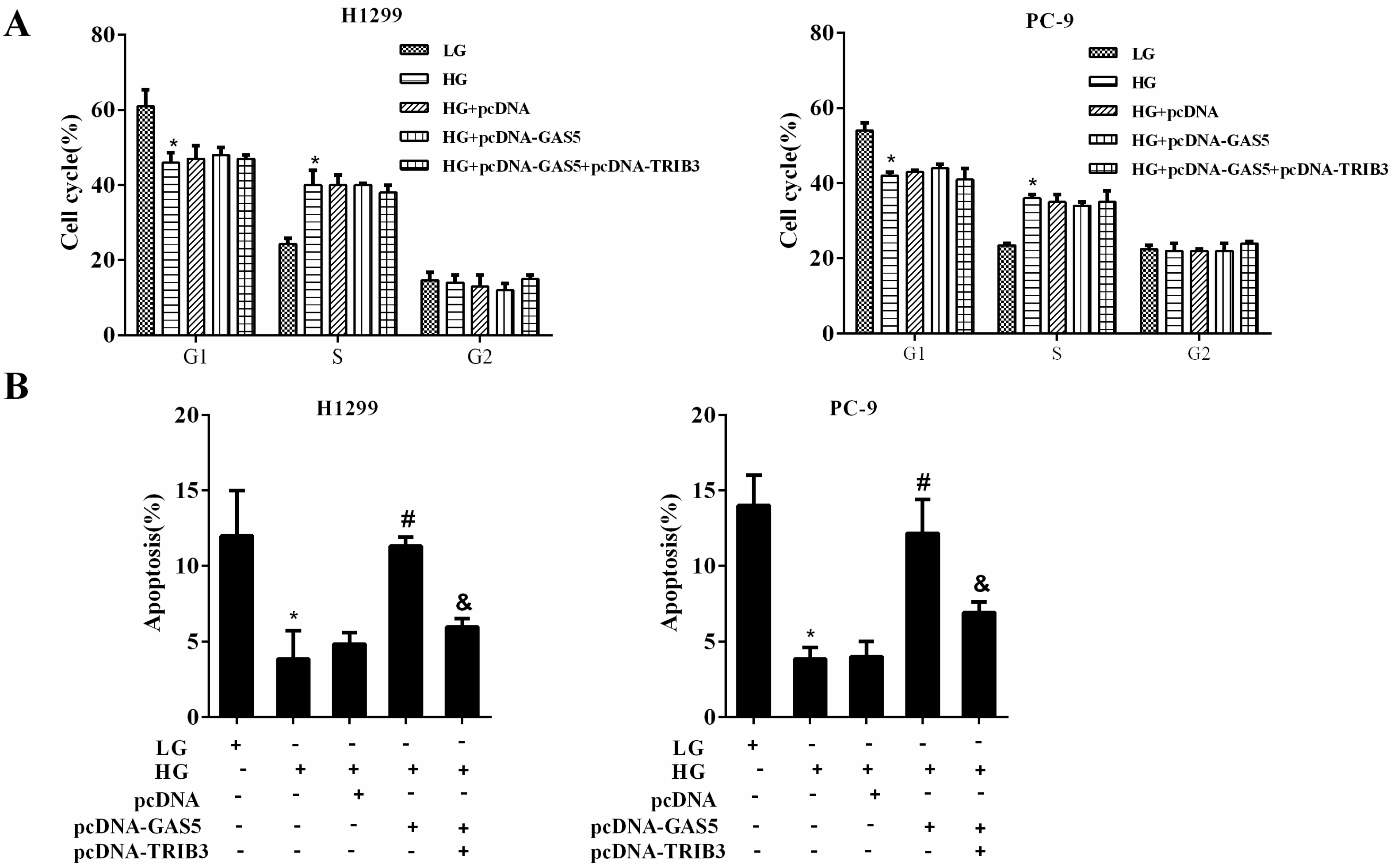
A
H1299
Cell cycle(%)
80
60
40
20
0
G1
S
G2
LG
HG
HG+pcDNA
HG+pcDNA-GAS5
HG+pcDNA-GAS5+pcDNA-TRIB3
PC-9
Cell cycle(%)
B
H1299
Apoptosis(%)
20
15
10
5
0
#
&
LG + - - - -
HG - + + + +
pcDNA - - + - -
pcDNA-GAS5 - - - + +
pcDNA-TRIB3 - - - - +
PC-9
Apoptosis(%)
LG + - - - -
HG - + + + +
pcDNA - - + - -
pcDNA-GAS5 - - - + +
pcDNA-TRIB3 - - - - +
